# Could Work Be a Source of Behavioural Disorders? A Study in Horses

**DOI:** 10.1371/journal.pone.0007625

**Published:** 2009-10-28

**Authors:** Martine Hausberger, Emmanuel Gautier, Véronique Biquand, Christophe Lunel, Patrick Jégo

**Affiliations:** Université de Rennes 1, UMR 6552 Ethologie Animale et Humaine, Unité Mixte de Recherche CNRS, Campus de Beaulieu, Rennes, France; University of Maryland, United States of America

## Abstract

Stress at work, as shown by a number of human studies, may lead to a variety of negative and durable effects, such as impaired psychological functioning (anxiety, depression…). Horses share with humans this characteristic of working on a daily basis and are submitted then to work stressors related to physical constraints and/or more “psychological” conflicts, such as potential controversial orders from the riders or the requirement to suppress emotions. On another hand, horses may perform abnormal repetitive behaviour (“stereotypies”) in response to adverse life conditions. In the present study, we investigated whether the type of work the horses are used for may have an impact on their tendency to show stereotypic behaviour (and its type) outside work. Observations in their box of 76 horses all living in the same conditions, belonging to one breed and one sex, revealed that the prevalence and types of stereotypies performed strongly depended upon the type of work they were used for. The stereotypies observed involved mostly mouth movements and head tossing/nodding. Work constraints probably added to unfavourable living conditions, favouring the emergence of chronic abnormal behaviours. This is especially remarkable as the 23 hours spent in the box were influenced by the one hour work performed every day. To our knowledge, this is the first evidence of potential effects of work stressors on the emergence of abnormal behaviours in an animal species. It raises an important line of thought on the chronic impact of the work situation on the daily life of individuals.

## Introduction

Stress at work, as shown by a number of human studies, may lead to a variety of negative and durable effects, such as impaired psychological functioning (anxiety, depression…), and be linked to adverse physical conditions such as gastro intestinal malfunction or musculoskeletal problems [1]. Although causation may be multi factorial, interpersonal stressors (conflicts, tensions…) account for more than 80% of the explained variance in daily mood [2]. According to Houtman et al. [3], musculoskeletal disorders may result not only from biomechanical solicitations at work but also from social stress. The requirement to suppress emotion in some categories of jobs has also been shown to be a significant source of work stress and may eventually have adverse effects on health [4], [5].

Horses share with humans this characteristic of working on a daily basis and have then “interpersonal” interactions not only with other working horses but also and mostly with a “boss” who is the human who manages or rides it [6]. Work sessions are based on training, using more often negative reinforcement or punishment than positive reinforcement [7]. Physical and emotional constraints depend also on the type of work performed. Negative consequences of some practices, leading to physical and behavioural resistances, open conflicts and tensions during the work sessions have been described for some time (e.g. [8]). Conflicting signals given by the rider (urge forward with the legs and keep restraining through the mouth bit) may lead the horse to frustration and neurosis [7]. Finally, horses are asked to suppress emotional reactions from their early stages of work on, as such reactions may be contrary to the performance expected (dressage competition) or considered dangerous for the rider (e.g. bucking) [9]. Few studies however question the possible durable effects of such work stressors (interpersonal conflicts, suppressed emotions, physical constraints) on the daily life of horses outside the work sessions.

Negative experiences linked to training may add to the effects of management style (e.g. social and spatial restrictions in the most widespread case where horses are in a box) and lead to chronic states where horses “switch off”, becoming unresponsive and apathetic [9], states described in humans in cases of work related burn out [10]. Abnormal repetitive behaviour, or stereotypic behaviour, is considered as clearly associated with poor welfare [11]–[13] and is suggested to be a way for animals to cope with an unfavourable stress-inducing environment (e.g. [14]–[15]). Indicators are pointing to an association between stereotypic behaviour and chronic stress [12].

Stereotypies in horses have been largely described and many factors may be involved, such as roughage availability (i.e. time spent foraging), diet, social deprivation, lack of exercise [16]–[20] as well as genetic susceptibilities (review in [21], [22]). Interestingly, although time spent performing stereotypies increases with time spent in stall [23], it may also increase with time spent working [24]. Stereotypies in thoroughbreds seem to increase around the age of 2, when training starts [25], whereas unbroken young horses show lower emotional reactions to a handling test than regularly trained show horses [26]. McGreevy et al. [23] observed differences in prevalence of stereotypies according to the type of work, dressage horses presenting the highest prevalence. These differences were attributed to differences in management practices. However different riding styles may impose different ranges of physical and psychological stressors on a horse [22], [8], that could explain these findings. Thus differences in the emotional reactions of horses (outside the working situation) in behavioural tests were observed according to the type of work [26]. Dressage training where horses have to perform restrained gaits and present a curved neck have more physical (and psychological?) constraints than jumping where horses are allowed more extended gaits and less pressure from the rider. A recent study showed that the “rollkür” posture (extreme neck curving) associated with some dressage practices was associated with more tail swishing, mouth opening and fear reactions than was observed in other horses [27].

In the present study, we investigated whether the type of work the horses are used for may have an impact on their tendency to show stereotypic behaviour (and its type) outside work. Observations performed on a large number of horses that differed only by the type of work (same living conditions, breed, sex) in their box showed that both the prevalence and types of stereotypies performed were related to the type of work the horses were used for. This is to our knowledge the first evidence in animals that work may be a source of chronic abnormal behaviour and raises new and general questions about the extra work consequences of stress at work.

## Results

### General findings

Observations of the behaviour of the horses in their box revealed that 65 out of the 76 subjects performed some type of stereotypy (0.83% to 36.67% of their time budget: −X = 5.488±6.81%). The proportion of horses involved did not differ between work groups (81 to 100%) (Table 1). This very high rate did reflect unsuitable environmental conditions [28, in prep.].

**Table 1:** Distribution of stereotypic horses in relation to type of work.

	Eventing	Jumping	Advanced school	Dressage	High school	Voltige	Total
**Total observed**	10	19	7	17	16	7	76
**Stereotypic horses**	10	17	6	15	13	4	65
**2 or more stereotypies**	1	3	0	4	6	1	15
**Licking/biting**	9	13	5	7	6	2	42
**Weaving**	0	0	1	1	1	0	3
**Head tossing/nodding**	1	4	0	5	6	1	17
**Cribbing/windsucking**	0	0	0	2	4	0	6
**Tongue Play**	6	14	4	10	10	4	48

Some horses performed two or more types of stereotypies: this tendency was observed mostly in dressage and high school (see definition in Appendix S1) horses. Repetitive licking and/or biting of substrates was observed mostly in eventing horses, whereas cribbing and windsucking occurred only in dressage and high school horses (Table 1). Times spent performing different types of stereotypies (Table 2) differed according to the type of work, especially for licking/biting (Kruskal-Wallis test, H_5_ = 12.66, P = 0.027), but such a tendency was also observed for cribbing and windsucking (H_5_ = 10.27, P = 0.068).

**Table 2:** Time spent performing different types of stereotypies (stereotypic horses only) according to type of work (% of scans, N = 120 scans per horses) (Mean±standard deviation).

	Eventing	Show jumping	Advanced school	Dressage	High School	Voltige
**N**	10	17	6	5	13	4
Weaving	0.00±0.00	0.00±0.00	0.14±0.34	0.44±1.72	0.06±0.23	0.00±0.00
Head tossing/nodding	0.08±0.26	0.31±0.60	0.00±0.00	1.67±4.35	2.18±4.57	0.21±0.42
Cribbing/windsucking	0.00±0.00	0.00±0.00	0.00±0.00	0.28±0.87	0.83±2.28	0.00±0.00
Tongue Play	2.17±2.73	1.85±2.07	1.67±1.58	3.24±5.59	1.60±1.34	1.88±1.58
Licking/biting	4.31±5.37	1.72±2.03	1.39±1.01	2.50±4.11	0.58±0.79	1.88±3.22

### Type of work and types of stereotypies: an overview (Figure 1)

The factorial correspondence analysis table crossed time spent performing each type of stereotypy (in columns) with each individual horse (in rows). Three factors accounted for 81.7% of the total inertia (variance). The first two axes accounted for 58.5% of the inertia. Table 3 summarizes the factor loadings of behavioural measures and qualitative predictors.

**Figure 1:**
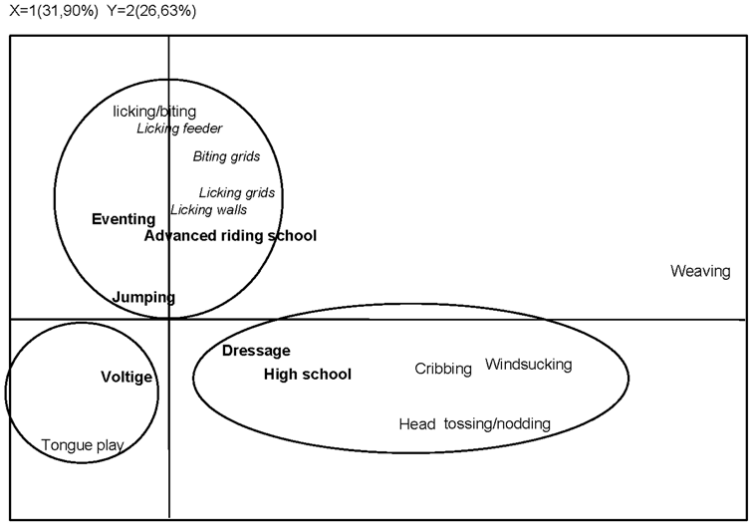
Graphical representation of the first two axes of the FCA performed on the two ways contingency tables: five stereotypies crossed by 65 horses from seven types of work. Each type of work is plotted as the barycenter of the horses working in that type of work.

**Table 3:** Factor loadings of the Factorial Correspondence Analysis (FCA) performed on the two ways contingency tables: five stereotypies crossed by 65 horses from seven types of work.

	Factor loadings of variables			
		F1	F2	F3
**Stereotypies**	Cribbing	187	9	31
	Tongue play	469	482	49
	Licking/biting	29	958	11
	Tossing/nodding	557	89	202
	Weaving	301	2	693
**Types of work**	Eventing	381	555	9
	Jumping	540	47	67
	Dressage	642	314	0
	High_school	462	187	27
	Advanced riding school	27	243	717
	Voltige	458	316	10

Factor loadings are the squared correlation coefficients between the variables and factors.

Axis 1 mainly opposed weaving, head tossing/nodding and windsucking/cribbing to tongue play and licking/biting, as well as high school and dressage horses to the others. Axis 2 opposed licking/biting to tongue play and head tossing/nodding, and to a lesser extent windsucking/cribbing, as well as eventing and instruction horses to high school, dressage and voltige horses. Three categories of horses associated with particular stereotypies emerged: dressage and high school horses, associated with windsucking/cribbing and head tossing/nodding; voltige horses associated with tongue play, and eventing, jumping and advanced riding school horses associated with repetitive licking/biting (Figure 1).

The third axis was mainly related to weaving and advanced riding school horses.

Given the emergence of these categories, further statistical analyses were performed to compare dressage/high-school horses (category 1, N = 28) to eventing/jumping/advanced-riding-school horses (category 2, N = 33). Voltige horses were too few for a separate comparison, but clearly showed more minor stereotypies than the other categories. It is interesting to note that they also spent more time lying down in the box than the other categories (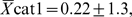 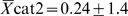, 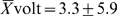 Kruskal Wallis H = 8.6 p = 0.01).

### Types of sterotypies and working categories (Figure 2)

Comparisons between categories revealed clear differences in the proportions of horses licking/biting (χ_1_^2^ = 3.94, P = 0.047) mainly performed by category 2 horses, or windsucking/cribbing (χ_1_^2^ = 7.17, P = 0.007) mainly performed by category 1 dressage and high-school horses, which also were more numerous to perform head tossing/nodding (χ_1_^2^ = 3.65, P = 0.056).

**Figure 2:**
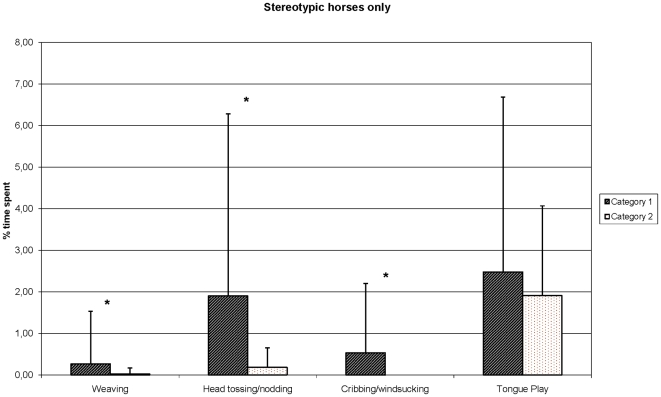
Mean (±standard deviation) percentage of time spent performing stereotypic behaviour in the box according to working category: Category 1 (N = 28): dressage and high school horses. Category 2 (N = 33): eventing, jumping and advanced riding school. *: statistically significant difference between the two groups, P<0.05 Mann Whitney U test. Note that despite the large variations in the percentage of time spent performing stereotypic behaviours by individual horses (see also table 2), differences between work categories are statistically significant, confirming its strong impact.

Time spent performing the different types of stereotypies also differed according to the work category: head tossing/nodding (Mann-Whitney U test, U = 216.5, N1 = 26, N2 = 24, P = 0.02) and windsucking/cribbing (Mann-Whitney U test, U = 234, N1 = 26, N2 = 24, P = 0.01) represented a larger part of the time budget of category 1 horses, whereas licking/biting were performed more frequently by category 2 horses (Mann-Whitney U test, U = 194, N1 = 26, N2 = 24, P = 0.02).

The two categories of horses also differed in more general aspects. Thus, more dressage/high-school horses tended to perform two or more stereotypies (χ_1_^2^ = 3.7, P = 0.056).

## Discussion

Observations of the behaviour of horses in their boxes revealed that the prevalence and types of stereotypies performed were related to the type of work they were used for. This is especially remarkable as all other factors of variations were controlled (same diet, same housing, one single site, one breed, one sex, no selection for the type of work: see methods). The daily one-hour working session appeared thus to have lasting effects on the remaining 23 hours horses spent in their stable.

This is to our knowledge the first evidence in animals that work may be a source of abnormal repetitive behaviour.

Our multivariate analysis, based on the occurrence of the different types of stereotypies, clearly divided types of work into three groups: jumping/eventing/advanced-riding-school, dressage/high-school, and voltige. Voltige horses appeared to be the least prone to stereotypies and performed relatively “mild types” such as tongue play, whereas dressage/high-school horses presented the highest incidence of stereotypies, as several of these horses performed two or more types of stereotypies. They also performed the “more serious” stereotypies (cribbing, windsucking, head shaking…). Finally, this analysis separated “minor types”, such as tongue play, repetitive licking/biting, from “more serious types” that were unexpectedly associated here. Weaving was performed by only a few horses and was separated from all other types of stereotypic behaviour.

This separation of the types of work into 3 categories on the basis of the type of stereotypies performed are especially interesting as they confirm that work characteristics (stressors?) are at stake. Dressage and high school both expect horses to restrain from expressing emotions and put a strong physical constraint on the movements. Moreover, cases where orders can be conflictual are more frequent here as the restricted gaits are often obtained by refraining movement through the reins and bit while pushing forward the horse through the legs [7].Therefore both physical and interactional stress can explain the high prevalence and types of stereotypies observed in these horses.

Jumping, eventing or instruction horses were trained more to take long strides while moving forward in a less ritualized posture. These horses performed more repetitive licking or biting of environmental structures. These activities are often considered to be early stages of stereotypy as they can be observed in foals weaned under unsuitable conditions [29], [19]. Whether these horses would develop more serious stereotypies with time appears unlikely as they remained under these conditions for at least one year and often more. Maybe they were reacting mainly to the general unsuitable conditions (social separation…) they were housed in.

An alternative explanation would be the demanding physical aspect of their work, which may have induced a search for elements missing in their diet.

Finally, voltige horses appeared the least prone to perform stereotypies and these were restricted mainly to tongue play. Voltige horses had been chosen for their quiet temperament and spent their work time turning in circles, with voice orders. “Interpersonal conflicts” with the human are rather limited as they are just required to keep regular and slow paces, while accepting humans to make movements on their backs. Their originally quieter temperament may also make them more resistant to possible work stressors as observed in humans (e.g. [30]). It is worth noting that, when working, these horses wore a reining device that kept their necks bent and their heads down. One could speculate that having their tongues out may reflect a resistance to their bits and associated apparatus that exert pressure on their mandibles.

According to Odberg [31], the emergence of abnormal behaviour could follow three steps: 1- trying to avoid a situation, 2- automatization of behaviour in the situation, 3- emancipation: that behaviour is performed independently of the situation. Some of the stereotypies observed, such as tongue play, may have developed this way, from trying to avoid bit pressure when being ridden to performing that behaviour in their boxes, away from the original constraint.

This possible lasting effect of strong bit action has been suggested for head shaking [32]. According to Cook [33], [34], the bit would be a risk factor of headshaking, as bit pressure could damage this region of the trigeminal nerve. Many riding horses present reactions in this region [35]–[37], one or the other branch of this nerve being hypersensitive. This sensitivity can be enhanced when horses have a hard permanent contact with their rider's hand and flexed cervical vertebrae in order to keep their heads down, as in many current dressage situations [8].

This would explain why headshaking and nodding were performed more often by dressage horses as for most of their working time they have to keep their necks flexed in restrained gaits (see also [27]).

These results strongly suggest therefore that work stressors are at stake in the emergence of the stereotypic behaviours observed. The unfavourable housing conditions (social and spatial restriction) outside the work sessions may have still emphasized their impact and the chronicity observed [9].

Although some work stressors involved here may be specific to equine work, others are clearly shared with other species, including humans: emotions suppression, interpersonal conflict, physical demands, lack of reward and negative future expectancy that are associated with depression in humans [30], [38], [5], [2].Thus, in a recent study, we could show that the use of negative reinforcement led to increased emotional expectancy on future actions [38].

The present study opens clearly new and further lines of thought about on one hand the causation of abnormal repetitive behaviours, on another hand the effects of work stressors not only on well known expressions of psychological disorders such as depression or burn out but also on the possible emergence of abnormal behaviour.

The riding situation may induce a hyper reactive state [7], [27] and the very controlled restricted locomotion allowed in dressage and especially high school horses associated with rapid transitions may explain an increase of reactivity, especially when bit pressure (see above) and spurs induce additional aversive stimulations [8]. The higher emotional responses of dressage horses in emotional tests [26] provide further support for this hypothesis. Collected gaits may also be physically very demanding and these difficulties may frustrate the horse, but also its rider who can transmit additional nervousness.

Further studies associating observations in working situations to behavioural observations in the box, are now required. For example, the finding that voltage horses, in overall less affected by stereotypies or at least major types of stereotypies, spend more time lying down could be a further indicator that they “relax” more easily in the box [39] and may be less affected by the rather barren and unstimulating environment provided by the box. Work type may affect how individuals perceive their daily environment, as in humans where daily mood is strongly affected by stress at work (2).

Experimental tests performed in indoor arenas in this same facility revealed that emotional reactions outside work differ greatly according to the type of work the horse are used for (Hausberger et al. in prep), showing that the impact of this activity is on the whole general state of the animal whether in box or in arena. Unfortunately, it was not possible at that time to realize physiological samplings on these highly valuable horses nor to get precise informations on potential repetitive health problems. This constitutes fascinating new line of research that needs now to be developed.

New results indicate that like in humans, musculoskeletal problems may arise from stress at work (Lesimple et al. in prep) and induce “bad moods” outside work (Fureix et al. subm.).

This first study raises important issues concerning welfare and the understanding of stereotypies. We showed that, for a variety of reasons (physical, emotional…), the limited time spent with humans might affect the remaining daily life of the horses. This may well be true for other situations such as handling, feeding, transporting animals. These results also raise the question of how different types of repetitive movements may develop. While some may be explained by lasting effects of physical constraints, others may emerge through chronic stress and be mediated by entero-gastric digestive pathologies [40]. Some stereotypies could possibly result from the automatization of some work [41].

## Materials and Methods

### 1) Animals and observation procedures

Seventy-six French Saddlebred horses were observed at the “Ecole Nationale d'Equitation” at Saumur (France) in November 1994. They were 6- to 15-year old geldings, were all housed under the same conditions in single boxes and were all ridden for one hour everyday (see Table 1). They were fed pellets and hay twice a day and had water ad libitum.

Each horse was observed for 5 minutes in its box and instantaneous scans [42] recorded its behaviour every 10 seconds. All horses were observed 4 times (yielding 20 minutes of observation and 120 scans for each horse). Observations were made during three periods: 8 to 11 am, 1 to 4 pm and 5 to 7 pm. As meals were distributed between 6.30 to 7 am and 4.15 to 6.30 pm. the three daily observation sessions included periods before and after meals (favourable for observing repetitive movements, [17], [43]).

All the observations in the boxes were made by the same observer (E. G.).

The horses were divided into six groups according to the type of work they were used for (see description in Appendix S1): eventing (n = 10), show jumping (n = 19), advanced riding school (n = 7), dressage (n = 17), high school (n = 16), voltige (n = 7) and then, in accordance to results, into 3 categories: eventing/show jumping/advanced school, dressage/high school and voltage. These categories differed only in terms of type of work as we ensured that 1) age did not differ (cat 1: 8.86±2.59 years, cat 2: 10.3±3.7 years, cat 3: 11.4±4.4 years, Kruskal Wallis test H = 4.15 p = 0.12); 2) diet was the same: commercial pellets provided by automatic feeder (general to the whole facility) to all horses, at the same feeding time, 4 times per day (6.30 am, 11.30 am, 4.15 pm, 6.30 pm), amounts determined only by size/weight; 3) all horses had been working in this type of work for 1 to 2 years at least; 4) all had arrived when 4 to 5 years old; 5) categories of horses were mixed in different locations and types of stables aver the facility. Apart from the 7 voltige horses, none of these horses had been selected for a particular type of work when they arrived, at the age of 4 or 5. French saddlebreds at that time were only selected for jumping and therefore there were no bloodlines selected for dressage, or high school in our sample that could explain the behavioural differences observed (see results). Moreover, several bloodlines were found in different work categories.

### 2) Terminology and behaviour observed

The stereotypic behaviours observed here correspond to those described in several previous studies (review in [22]) and all consisted in functionless repetitive movements [12].

#### Weaving

obvious lateral swaying, movement of head, neck, forequarters and sometimes hindquarters.

#### Cribbing and windsucking

when cribbing, the horse grasps a fixed object with its incisors, pulls backwards and draws air into its oesophagus. Windsucking is similar but no object is grasped.

#### Head shaking and nodding

repetitive bobbing of head up and down or recurrent and sudden bouts of head tossing.

#### Tongue play

the horse sticks out its tongue and twists it in the air.

In addition to the “more classical” stereotypies we recorded repetitive licking/biting (walls, grids, feeder…) movements as additional abnormal repetitive behaviours.

### 3) Statistical analyses

Two statistical approaches were used: a Factorial Correspondence Analysis (FCA) and non-parametric statistical tests.

Factorial analysis is a descriptive but very informative approach yielding a simultaneous plot of both groups of variables tested (here time spent performing stereotypies and horses characterized by their type of work) and a visualisation of their relationship.

The quantitative data used were the occurrence of the stereotypies in the scans obtained for each horse.

Non-parametric statistical tests were used, as normality of data was not ensured: χ^2^ tests compared the numbers of animals performing stereotypies between groups. Mann-Whitney and Kruskal-Wallis non-parametric tests compared times spent performing different stereotypic activities between groups.

## Supporting Information

Appendix S1 (0.03 MB DOC)
