# Supplementary material for: Could Work Be a Source of Behavioural Disorders? A Study in Horses
Source: PLoS One. 2009 Oct 28;4(10):e7625. doi: 10.1371/journal.pone.0007625 (PMC2763287; doi:10.1371/journal.pone.0007625)
Supplement: Appendix S1 — (0.03 MB DOC) [file pone.0007625.s001.doc]

## Appendix S1

### Type of Work

.

**Show jumping:** horses are used to jump over a series of artificial obstacles in a limited time while being ridden by experienced riders.

**Dressage:** horses have to perform figures in an arena of a given area, such as circles, stops, and changing pace at given points in the arena, showing docility to riders' orders. Their paces are very controlled and neck is maintained flexed.

**High school riding:** horses perform the same tasks as in dressage but in addition must perform higher technical tasks and elevated paces (stamping, etc.).

**Eventing:** horses have to perform some dressage, jumping over artificial and natural obstacles while being ridden by experienced riders.

**Voltige:** horses have to turn in circles with their trainer in the middle using a long lunge. The horses have to maintain a regular pace (mostly slow canter) while riders jump onto their backs and may perform different exercises (e.g., standing on the horse's back). Orders are mainly given by voice.

**Advanced riding school:** horses were used to train experienced riders in order to prepare them to become instructors. They performed a variety of tasks, mostly jumping, eventing and training the riders’seats.
